# Effects of Feeding Rates on Growth Performance and Liver Glucose Metabolism in Juvenile Largemouth Bronze Gudgeon (*Coreius guichenoti*)

**DOI:** 10.3390/ani14172466

**Published:** 2024-08-25

**Authors:** Pei Chen, Huantao Qu, Jing Yang, Yu Zhao, Xu Cheng, Wei Jiang

**Affiliations:** Hubei Key Laboratory of Three Gorges Project for Conservation of Fishes, Chinese Sturgeon Research Institute, China Three Gorges Corporation, Yichang 443100, China; qu_huantao@ctg.com.cn (H.Q.); yang_jing7@ctg.com.cn (J.Y.); zhao_yu@ctg.com.cn (Y.Z.); cheng_xu2@ctg.com.cn (X.C.)

**Keywords:** largemouth bronze gudgeon, growth performance, glycolysis, gluconeogenesis, glycogen

## Abstract

**Simple Summary:**

Currently, less information regarding the impact of feeding rates on glucose metabolism in largemouth bronze gudgeon has been reported. In this study, we investigated the effects of feeding rates on growth performance, hepatic glycolysis, gluconeogenesis, and glycogen metabolism in juvenile largemouth bronze gudgeon. The results demonstrated that a feeding rate exceeding 3% significantly increased liver glycolysis and glycogen synthesis in juvenile largemouth bronze gudgeon, subsequently accelerating hepatic glycogen and lipid accumulation, which ultimately induced fatty liver formation.

**Abstract:**

The experiment was conducted to investigate the effects of feeding rates on growth performance, liver glycolysis, gluconeogenesis, glycogen synthesis, and glycogen decomposition in juvenile largemouth bronze gudgeon (*Coreius guichenoti*). A total number of 600 fish were randomly distributed into 12 cylindrical plastic tanks with 50 fish per tank and triplicate tanks per treatment. Fish were fed with 2%, 3%, 4%, and 5% feeding rates (body weight per day) three times day^−1^ for 8 w. The results indicated that the feeding rates significantly increased the body weight, weight gain rate, and specific growth rate (*p* < 0.05), while showing no significant effects on the condition factor and survival rate (*p* > 0.05). The feed conversion ratio was significantly enhanced by the feeding rate (*p* < 0.05), although no significant differences were observed when the feeding rate exceeded 3% (*p* > 0.05). The plasma glucose levels in the 4% and 5% groups were significantly higher than those in the 2% and 3% groups. Compared with other groups, the 5% group significantly increased the crucial rate-limiting enzyme activities and mRNA levels of glycolysis (PFKL and PK) (*p* < 0.05), while showing no significant differences on enzyme activities (PC, PEPCK, and G6P) and mRNA (*pepck* and *g6p*) levels of gluconeogenesis (*p* > 0.05). In addition, the mRNA levels of hepatic *glut2* and *glut4* in the 5% group reached the highest levels (*p* < 0.05). When the feeding rate exceeded 3%, hepatic glycogen and lipid accumulation were significantly increased, leading to a fatty liver phenotype. Meanwhile, the mRNA level of liver glycogen synthetase (*gysl*) was significantly increased (*p* < 0.05), while no significant difference was observed in glycogen phosphorylase (*pygl*) (*p* > 0.05). In summary, under the conditions of this study, a feeding rate exceeding 3% significantly accelerated hepatic glycogen and lipid accumulation, which ultimately induced fatty liver formation.

## 1. Introduction

Largemouth bronze gudgeon (*Coreius guichenoti*) was historically the dominant species in the upper reaches of the Yangtze River in China. This typical riverine migratory fish spawns floating eggs, completing its entire life cycle in flowing water habitats [1,2]. In recent years, hydroelectric development in the middle and lower reaches of the Jinsha River and Yalong River has significantly altered the habitat, diet, and reproductive ecology of the largemouth bronze gudgeon. These changes pose serious threats to its survival and reproduction, resulting in a rapid decline in its wild population [3]. In 2021, the “List of National Key Protected Wild Animals” officially designated the largemouth bronze gudgeon as a national second-class protected species. Despite numerous conservation policies and ecological compensation measures implemented by government departments and conservation agencies, the natural population of largemouth bronze gudgeon continues to decline [4]. Therefore, there is an urgent need to strengthen the protection and restoration of largemouth bronze gudgeon resources.

With advancements in artificial culture, domestication, and breeding technology, a sustainable breeding population of largemouth bronze gudgeon has gradually been established [4,5]. However, the weak stress resistance and high mortality rate of juvenile largemouth bronze gudgeon are significant factors limiting the scale of artificial breeding [6,7]. Feeding rates have a substantial impact on the growth and feed utilization efficiency of fish. Providing adequate and balanced nutritional feed is crucial for promoting fish growth and reducing disease incidence [8]. Optimal feeding rate can promote growth, improve feed efficiency, and reduce breeding costs in fish such as the tilapia (*Oreochromis niloticus*) [9], longsnout catfish (*Leiocassis longirostris*) [10], and Yangtze sturgeon (*Acipenser dabryanus*) [11]. Both underfeeding and overfeeding can lead to poor growth and decreased survival rates in fish [12,13]. The liver plays a vital role in glucose synthesis and metabolism in fish, which is essential for their growth, immunity, and stress resistance [14,15]. Generally, excessive feeding results in a nutrient surplus, with excess nutrients stored in the liver as glycogen, which, if excessive, can convert to hepatic lipids [15,16].

Currently, there is limited research on the nutritional composition of the body, hepatopancreas, muscle, and gonadal tissues of largemouth bronze gudgeon [1,17,18], and no studies are focusing on its nutritional requirement. As a primarily carnivorous omnivorous fish, largemouth bronze gudgeon requires high-nutritional compound feed for large-scale cultivation [19]. Presently, commercial compound feed is predominantly utilized in artificial breeding. Previous studies indicated that feeding three times day^−1^ at a 3% feeding rate optimized growth, intestinal digestion, and liver health in juvenile largemouth bronze gudgeon [7,20]. In practice, farmers typically adopt higher feeding rates to enhance the growth performance of fish. However, as we all know, increased feeding rates lead to elevated hepatic lipid synthesis and reduced lipolysis, resulting in liver lipid accumulation and fatty liver development, which negatively impacts fish health. [21]. Up to now, there is no information on how feeding rates affect glucose metabolism in largemouth bronze gudgeon. This experiment tested 2%, 3%, 4%, and 5% feeding rates to investigate their effects on growth performance, hepatic glycolysis, gluconeogenesis, and glycogen metabolism in juvenile largemouth bronze gudgeon. The results of this study will provide a scientific basis for the healthy cultivation of largemouth bronze gudgeon.

## 2. Materials and Methods

### 2.1. Experimental Design and Breeding Management

Artificially propagated and cultured juvenile largemouth bronze gudgeon were obtained from Treasure Endemic Fish Breeding and the Releasing Station of Jinsha River Xiluodu Xiangjiaba Hydropower Station (Yibin, China). A total of 600 healthy juvenile largemouth bronze gudgeon (initial body weight of 4.97 ± 0.11 g), approximately 6–7 cm in length, were selected and divided into four groups. The fish were raised in 12 round fiberglass tanks (height 75 cm × diameter 50 cm; water depth: 70 cm), each tank holding 50 juveniles in triplicate. During the rearing period, the four groups were fed at 2%, 3%, 4%, and 5% feeding rates (apparent satiation feeding rate is 5% [20]), respectively, 3 times day^−1^ at 09:00, 13:00, and 17:00. The feed used was microparticle compound feed for fish (particle size 1.0 mm) produced by Shandong Shengsuo Feed Technology Co., Ltd., (Yantai, China) with the main ingredients including imported special fish meal, Antarctic krill meal, refined fish oil, lecithin, choline chloride, vitamins and provitamins, and mineral elements. The nutritional composition was as follows: crude protein 52.66%, crude fat 14.33%, starch content 10.6%, and moisture 6.92%. The feeding amount was adjusted every two weeks based on the weight of the experimental fish. The water temperature in the recirculating water system ranged from 16 to 22 °C, with a light cycle of 12:12 h, and dissolved oxygen levels maintained at ≥7.0 mg/L.

### 2.2. Sample Collection and Growth Index Calculation

After 8 weeks of rearing, all experimental fish were starved for 24 h, then weighed and counted. Six fish were randomly taken from each tank, anesthetized with 50 mg/L MS-222 (ethyl 3-aminobenzoate methanesulfonate), and measured for body length and weight. Blood was rapidly sampled from the caudal vein, centrifuged (4000× *g*, 10 min, 4 °C) to obtain plasma for glucose analysis. Twelve liver samples near the bile duct in each treatment were collected, with part of it fixed in a 4% paraformaldehyde solution for hematoxylin–eosin (H.E.) and periodic acid–Schiff (PAS) staining, while the remainder was quickly frozen in liquid nitrogen for physiological index and gene expression analysis.

The growth indices were calculated using the following formulas: weight gain rate (WGR, %) = 100 × (final average weight − initial average weight)/initial average weight; specific growth rate (SGR, %/d) = 100 [Ln(final average weight) − Ln(initial average weight)]/56; feed conversion ratio (FCR) = 100 × feed intake/(final weight − initial weight); condition factor (CF, g/cm^3^) = 100 × final weight/(final length)^3^; and survival rate (SR, %) = 100 × (final fish number/initial fish number).

### 2.3. Liver Physiological Parameters Detection

The liver was ground using liquid nitrogen, then 9 volumes of PBS were added and shaken. The mixture was centrifuged at 4000 r/min for 10 min at 4 °C, and the supernatant was collected for physiological index determination. Total protein (TP), glucose, and liver glycogen detection kits were purchased from the Nanjing Jiancheng Bioengineering Institute (Nanjing, China). Kits for detecting glucokinase (GK), phosphofructokinase liver (PFKL), pyruvate kinase (PK), pyruvate carboxylase (PC), phosphoenolpyruvate carboxykinase (PEPCK), and glucose-6-phosphatase (G6P) were purchased from Shanghai Enzyme-linked Biotechnology Co., Ltd. (Shanghai, China). The liver physiological parameters were all measured with a microplate reader (PowerWave XS2, BioTek, Winooski, VT, USA) following the instructions provided with the reagent kits.

### 2.4. Liver Tissue Section Examination

Simply, liver tissues were fixed in a 4% paraformaldehyde solution for 48 h, dehydrated in graded ethanol concentrations, embedded in paraffin wax, and cut into 6 µm sections. Subsequently, liver sections were stained with H.E. and PAS staining. After staining, the sections were observed with detailed steps according to Chen et al. [22] and photographed by a light microscope with a computerized image system (Olympus, DP73, Tokyo, Japan). The relative areas of hepatic lipid drops and glycogen particles in H.E. staining and PAS staining, respectively, were quantified by image processing software (Image J v.1.8.0.112) according to Zhu et al. [7].

### 2.5. RNA Isolation, Reverse Transcription, and Quantitative Real-Time PCR Analysis

According to the instructions of the TRNzol Universal total RNA extraction reagent (Tiangen, Beijing, China), mRNA was extracted from liver tissues. The isolated RNA was quantified using Ultramicro spectrophotometer NanoDrop 2000c (Thermo Sci, Waltham, MA, USA), and the RNA quality was assessed through electrophoresis using a 1.5% (*w*/*v*) agarose gel. The first-strand cDNA was synthesized from lμg of the total RNA using the FastKing one-step reverse transcription kit (Tiangen, China). The cDNA served as the template. The 2 × SYBR qPCR mix (High ROX) (Aidlab, Beijing, China) was used for real-time fluorescent quantitative detection with the AB Step One Plus Real-Time PCR instrument (Thermo Fisher, Waltham, MA, USA). The reaction volume (20 μL) contained 10 μL of 2 × SYBR, 2 μL of the cDNA template, 1 μL of each 10 μM specific primer, and 6 μL of RNA-free water. The amplification conditions were as follows: 95 °C for 3 min of pre-denaturation, followed by 95 °C for 10 s of denaturation, and 60 °C for 35 s of annealing and extension, for a total of 35 cycles. 

The genes of the largemouth bronze gudgeon were obtained from RNA-seq data (GenBank accession no. PRJNA1149284). EF1α (elongation factor 1α), a housekeeping gene, was used as an endogenous reference to normalize the template amount [7]. Specific primers of target genes were designed using Primer Premier 5.0 software and presented in Table 1. The specificity of the reaction product was validated by the observation of a single melt peak. Each sample was run in three technical replicates, and the relative expression levels of the target genes were calculated using the 2^−△△CT^ method.

### 2.6. Data Statistics and Analysis

The experimental results are presented as mean ± standard error and analyzed by SPSS 26.0 software (USA). Following the assessment of the homogeneity of variance, all data were analyzed using a one-way ANOVA, followed by Duncan’s post hoc test with *p* < 0.05 indicating statistically significant differences. Independent samples *t*-tests were used to determine significant differences between the two liver phenotypes. The figures in the study were generated using GraphPad 9.0 software (USA).

## 3. Results

### 3.1. Effects of Feeding Rates on the Growth Performance

The effects of feeding rates on the growth performance of juvenile largemouth bronze gudgeon are shown in Table 2. The final weight, weight gain rate, and SGR significantly increased with higher feeding rates (*p* < 0.05). The feeding rate significantly increased the FCR (*p* < 0.05), but there was no significant difference observed beyond the 3% group (*p* > 0.05). No significant effects on CF and SR were found among the groups (*p* > 0.05).

### 3.2. Effects of Feeding Rates on Liver Tissue Sections and Glycogen Synthesis

**Figure 1:**
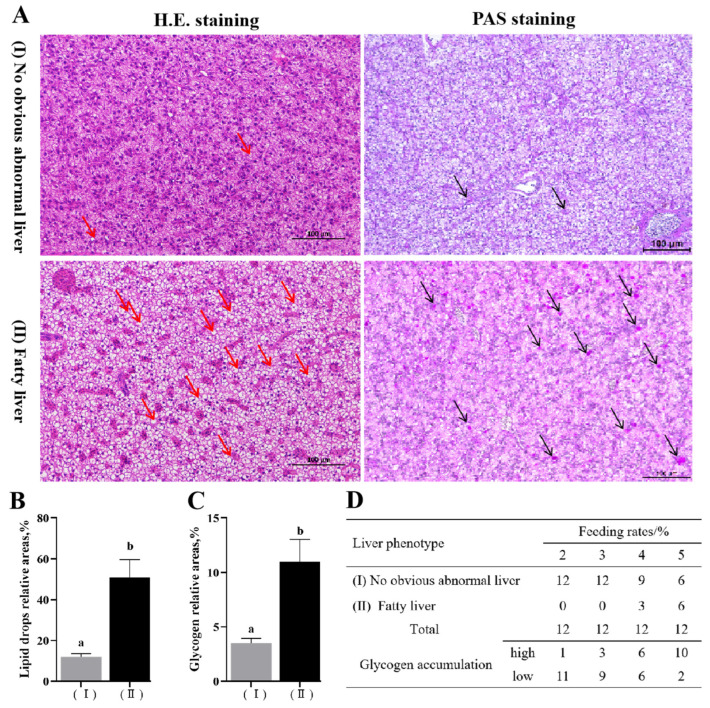
Hepatic histopathological analysis of largemouth bronze gudgeon. (**A**) Two phenotypes of hepatic histopathological examination: (I) no obvious abnormal phenotype; and (II) fatty liver phenotype. The red arrow represents the lipid drops. The pink spot in the section represented the glycogen particles (shown in black arrows). (**B**) The relative areas of hepatic lipid drop by H.E. staining. (**C**) The relative areas of hepatic glycogen by PAS staining. (**D**) The statistical results of two phenotypes in each feeding rate group. Data columns with different lowercase letters in the figure indicate significant differences between no obvious abnormal phenotype and fatty liver phenotype (*p* < 0.05).

**Figure 2:**
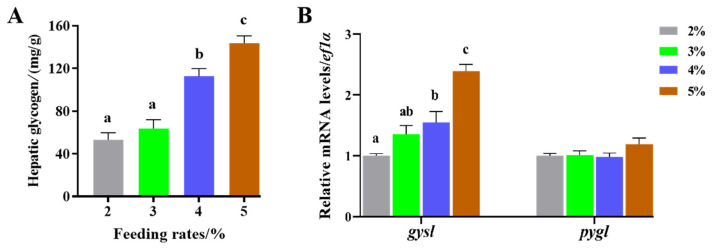
Effects of feeding rates on (**A**) hepatic glycogen contents and (**B**) gene expression related to hepatic glycogen synthesis and decomposition in juvenile largemouth bronze gudgeon. Data columns with different lowercase letters in the figure indicate significant differences between groups (*p* < 0.05).

Two distinct liver phenotypes were identified through H.E. and PAS staining (Figure 1A): (I) No obvious abnormal phenotype, characterized by liver cell contours and nuclei that were very clear, with few lipid droplets and glycogen granules. (II) Fatty liver phenotype, where liver cell contours and hepatic cords were unclear, and significant lipid vacuoles appeared in the liver cells, accompanied by an increase in the size and number of lipid droplets, as well as heightened glycogen accumulation (Figure 1B,C). The proportion of fatty liver phenotype escalated with increasing feeding rate, reaching 50% (6/12) in the 5% group (Figure 1D). Additionally, liver glycogen content significantly increased with the feeding rate (*p* < 0.05), with no significant difference between the 2% and 3% groups (Figure 2A). Compared with the 2% and 3% groups, the mRNA levels of the glycogen synthesis gene *gysl* in the 4% and 5% groups were significantly upregulated with the feeding rates (*p* < 0.05), while no significant differences were observed in the mRNA levels of the glycogen breakdown gene *pygl* (*p* > 0.05) (Figure 2B).

### 3.3. Effects of Feeding Rate on Plasma Glucose, Liver Glycolysis, and Gluconeogenesis Metabolism-Related Enzyme Activities and Gene Expression

**Figure 3:**
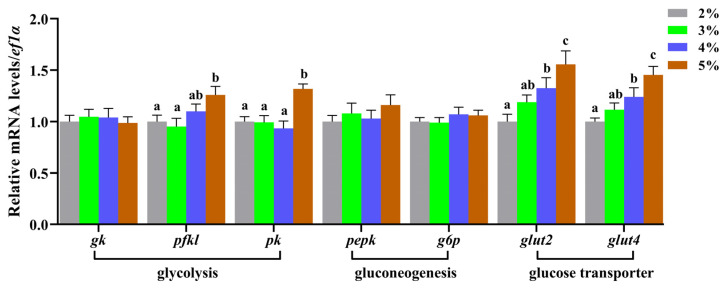
Effects of feeding rates on gene expression related to hepatic glycolysis and gluconeogenesis in juvenile largemouth bronze gudgeon. Data columns with different lowercase letters in the figure indicate significant differences between groups (*p* < 0.05).

The effects of feeding rate on plasma glucose, liver metabolism-related enzyme activities, and gene expression in juvenile largemouth bronze gudgeon are summarized in Table 3 and Figure 3. As the feeding rate increased, the plasma glucose level gradually rose. The plasma glucose levels in the 4% and 5% groups were significantly higher than those in the 2% and 3% groups (*p* > 0.05). Liver glucose levels increased with the feeding rate and then decreased, peaking in the 3% group, while the TP content showed no significant difference (*p* > 0.05). Compared with the 2% group, only the activities and mRNA levels of key rate-limiting glycolytic enzymes PFKL and PK were significantly increased in the 5% group (*p* < 0.05), whereas GK activity and mRNA levels did not exhibit significant differences (*p* > 0.05). Furthermore, the feeding rate had no significant effect on the activities of key rate-limiting gluconeogenesis enzymes PC, PEPCK, and G6P, nor on the mRNA levels of *pepck* and *g6p* (*p* > 0.05). The mRNA levels of glucose transporter-related genes *glut2* and *glut4* were significantly upregulated when increasing the feeding rate (*p* < 0.05), reaching the highest expression levels in the 5% group.

## 4. Discussion

### 4.1. Effects of Feeding Rate on the Growth Performance

Excessive feed energy, unreasonable protein–energy ratio, high-carbohydrate and high-fat diets, and excessive feeding rates can cause liver glycogen synthesis to exceed the required amount, leading to glycogen and lipid accumulation, which forms fatty liver and consequently damages fish health [14,15]. This study found that the final body weight, WGR, and SGR of juvenile largemouth bronze gudgeon significantly increased with a higher feeding rate. Similar results have also been reported in cuneate drum (*Nibea miichthioides*) [23], European sea bass (*Dicentrarchus labrax*) [24], and Ussuri catfish (*Pseudobagrus ussuriensis*) [8]. Previous research had indicated that when the feeding rate exceeds 3%, the liver of juvenile largemouth bronze gudgeon tended to accumulate lipid and induced fatty liver, but their growth performance did not exhibit a downward trend [20,21], consistent with the results of this study. 

The condition factor is an important morphological indicator to measure fish growth quality. Typically, a high feeding rate can enhance nutrient absorption, while excessive nutrient/energy intake can lead to an increase in the condition factor [10]. Conversely, this study found that the feeding rate had no significant effect on the condition factor. Under natural conditions, largemouth bronze gudgeon typically inhabit fast-flowing water areas and exhibit fast swimming behavior, which necessitates a high energy expenditure [17,20]. Furthermore, the lipid content of wild largemouth bronze gudgeon ranges from 7.11% to 10.96% for the whole fish and can reach up to 22% in the liver, which is higher than the normal levels observed in most fish [17]. Hepatic transcriptome analysis of artificially bred largemouth bronze gudgeon parents and offspring revealed that differentially expressed genes were significantly enriched in fatty acid digestion, absorption, metabolism, and synthesis pathways [25]. Therefore, it can be inferred that largemouth bronze gudgeon possesses a robust ability to accumulate and utilize lipids to meet their energy demands [21,26]. From the above results, it can be concluded that a high feeding rate did not lead to a decline in growth performance or an increase in the condition factor of largemouth bronze gudgeon, which may be related to their well-developed hepatopancreas and habitat preferences [1,20]. Consequently, based on growth performance and survival rates, we speculate that largemouth bronze gudgeon can adapt to the increase in high feeding rates to protect against growth decline from hepatic lipid accumulation. 

In addition, this study found that the feed efficiency of largemouth bronze gudgeon exhibited an upward trend with increasing feeding rate, followed by stabilization. This observation aligns with findings in loach (*Misgurnus anguillicaudatus*) [27], pufferfish (*Takifugu rubripes*) [28], and largemouth bronze gudgeon [20], which suggested that a high feeding rate might reduce the time that feed remains in the fish’s digestive tract, hindering complete digestion and absorption, and leading to a higher FCR. In this study, the high feed efficiency in the high feeding rate group may also be related to the fact that largemouth bronze gudgeon inhabits fast-flowing water areas, necessitating substantial energy expenditure.

### 4.2. Effects of Feeding Rate on Glucose Metabolism

As the center hub for glucose metabolism and production, the liver plays a crucial role in maintaining glucose homeostasis [15]. Generally, when the feeding rate is excessively high (excess nutrient intake), fish can alleviate hyperglycemic stress by enhancing glycolysis and hepatic glycogen synthesis, while reducing gluconeogenesis [16,22]. Glucose cannot directly enter cells to exert its functions; it requires glucose transporters (GLUT) on the cell membrane for cellular entry and subsequent metabolic participation [29]. Among these, GLUT2 and GLUT4 facilitate the absorption of glucose by cells, playing significant roles in regulating the body’s blood glucose balance [30]. Zhao et al. [31] found that the upregulated *glut2* mRNA levels in the hepatopancreas of white shrimp (*Litopenaeus vannamei*) accelerate glucose entry into cells. Furthermore, Yang et al. [32] demonstrated that insulin can upregulate the mRNA levels of *glut4* in the muscle of carp (*Cyprinus carpio* L.), promoting plasma glucose absorption. In the present study, when the feeding rate exceeds 3%, the mRNA levels of *glut2* and *glut4* in the liver of largemouth bronze gudgeon were significantly upregulated, indicating that a high feeding rate accelerated hepatic glucose absorption. Additionally, at feeding rates above 3%, the hepatic *gysl* mRNA levels were significantly upregulated, while the *pygl* mRNA levels showed no significant difference, resulting in a significant increase in the hepatic glycogen content. Consequently, in the group with a feeding rate exceeding 3%, the glucose content in the liver of largemouth bronze gudgeon decreases.

Generally, excessive nutrients are stored in the form of hepatic glycogen, and when hepatic glycogen levels become excessive, they are converted into hepatic lipids [15,16]. This study found that as the feeding rate increases, the proportion of hepatic fatty liver phenotype gradually rises, reaching as high as 50% in the 5% feeding rate group. This finding aligns with Zhao et al. [20], which indicated that a high feeding rate can lead to increasing hepatic lipid synthesis and decreasing lipolysis in juvenile largemouth bronze gudgeon, resulting in hepatic lipid accumulation, and ultimately inducing fatty liver formation [21]. Moreover, excessive hyperglycemia can provide NADPH or carbon skeletons for de novo lipid synthesis, thereby promoting lipid accumulation [14,16]. Therefore, largemouth bronze gudgeon may alleviate hyperglycemic stress caused by high feeding rates by storing lipids in the liver. However, further research is warranted to determine whether this hepatic lipid accumulation leads to fatty liver disease.

After glucose enters cells, it undergoes complete oxidation and decomposition through glycolysis and the tricarboxylic acid cycle, providing energy for the organism’s life activities. GK, PFKL, and PK are key rate-limiting enzymes of glycolysis, while PC, PEPCK, and G6P are key rate-limiting enzymes of gluconeogenesis. Changes in their activities play an important role in maintaining the rate of glucose metabolism [29]. Currently, there are limited studies on the effects of feeding rate on glucose metabolism in fish. He et al. [11] found that compared with the 3% feeding rate group, the 5% group significantly increased the hepatic glycogen content in juvenile Yangtze sturgeon, along with a notable increase in the activity of the key glycolytic enzyme HK (hexokinase), while G6PDH (glucose-6-phosphate dehydrogenase) activity significantly decreased. Additionally, G6P activity showed no significant difference. Previous studies have demonstrated that fish can respond to environmental changes (stress) by regulating glucose metabolism, thereby maintaining internal and external homeostasis. Particularly after excessive feeding or at a high feeding rate, fish can maintain plasma glucose balance by enhancing glycolysis and reducing gluconeogenesis [22,33]. For instance, in response to excessive feeding, largemouth bass (*Micropterus salmoides*) [21] and rainbow trout (*Oncorhynchus mykis*) [34] could mitigate hyperglycemic stress by upregulating the mRNA levels of hepatic glycolysis-related genes and downregulating the mRNA levels of gluconeogenesis-related genes. Chen et al. [22] reported that short-term excessive feeding significantly elevated the transcription levels and enzyme activities of the key rate-limiting enzymes PCK and G6Pase in the liver. This abnormal elevation in gluconeogenesis can lead to disorders in glucose metabolism in fish, damaging fish health. This study found that a 5% feeding rate can significantly increase the activities and mRNA levels of the key rate-limiting enzymes PFKL and PK in glycolysis, while also significantly affecting the activities of PC, PEPCK, and G6P, as well as the mRNA levels of *pepck* and *g6p*. This indicated that largemouth bronze gudgeon can actively respond to hyperglycemic stress induced by a high feeding rate by enhancing glycolysis and stabilizing gluconeogenesis.

## 5. Conclusions

In this study, a feeding rate exceeding 3% enhanced hepatic glycolysis and glycogen synthesis in largemouth bronze gudgeon, resulting in hepatic glycogen and lipid accumulation, which ultimately induced fatty liver formation. However, further research is required to ascertain whether the fatty liver induced by lipid accumulation harms fish health.

## Figures and Tables

**Table 1:** Primer sequences used in this study.

Genes	Forward Primer (5′-3′)	Reverse Primer (5′-3′)	Products (bp)
*ef1α*	TGGGTGTTGGACAAACTGAA	CAACACCACCAGCAACAATC	190
*gk*	GTCCCCATATCAGGGTGTCTT	CAACCGTTGTCAGAAGTCCAT	163
*pk*	ACTGGACACCAAAGGACCAG	GCTGGGATAATCCAACCAGA	157
*pfkl*	AGACTGCAGAAAGGGCAAAA	TTCTCTGCGGAAGGTCTTGT	154
*pepck*	ACCTGCACCTGGAATCAAAC	ACACACCATGACGCCAGTTA	236
*g6p*	TTCTCGTCTCTGAACCGTGAT	GAACAGTGGGAAGAGGGAAAC	163
*glut2*	CAGTTGCAACACCCAGCTAA	GGGCAGACGAACTCTCACTC	243
*glut4*	CCATGCCAATGATGAAGTTG	TGACAGGAGACTGTGCCATC	193
*gysl*	TTGCATAAATGGCCCTCTTC	CCTGCCAAAACCAACAACTT	199
*pygl*	TCTTTGACCAGCGTGAAGTG	CTCGGTGTAACCGGTGATCT	153

*ef1α*, elongation factor 1α; *gk*, glucokinase; *pk*, pyruvate kinase; *pfkl*, phosphofructokinase liver; *pepck*, phosphoenolpyruvate carboxykinase; *g6p*, glucose-6-phosphatase; *glut2*, glucose transporter type 2; *glut4*, glucose transporter type 4; *gysl*, glycogen synthase liver form; *pygl*, glycogen phosphorylase liver.

**Table 2:** Effects of feeding rates on growth performance in juvenile largemouth bronze gudgeon.

	Feeding Rates (%)				ANOVA *p* Value
	2	3	4	5	
initial body weight (g)	4.95 ± 0.12	5.01 ± 0.11	4.93 ± 0.08	4.98 ± 0.10	0.898
final body weight (g)	8.61 ± 0.26 ^a^	10.68 ± 0.37 ^b^	12.59 ± 0.43 ^c^	14.17 ± 0.35 ^d^	<0.001
WGR (%)	73.94 ± 4.23 ^a^	113.17 ± 4.54 ^b^	155.38 ± 5.32 ^c^	184.54 ± 5.65 ^d^	<0.001
SGR (%/d)	0.99 ± 0.08 ^a^	1.35 ± 0.07 ^b^	1.67 ± 0.08 ^c^	1.86 ± 0.06 ^d^	<0.001
FCR	0.46 ± 0.02 ^a^	0.53 ± 0.01 ^b^	0.55 ± 0.02 ^b^	0.54 ± 0.01 ^b^	0.012
CF (g/cm^3^)	1.42 ± 0.10	1.49 ± 0.08	1.48 ± 0.08	1.45 ± 0.06	0.905
SR (%)	97.33 ± 0.67	98.67 ± 0.67	98.67 ± 1.33	96.67 ± 0.67	0.344

In the same column, values with different small letter superscripts mean significant difference (*p* < 0.05), and the same applies below.

**Table 3:** Effects of feeding rates on plasma glucose and enzyme activity related to hepatic glycolysis and gluconeogenesis in juvenile largemouth bronze gudgeon.

	Feeding Rates (%)				ANOVA *p* Value
	2	3	4	5	
Hematological parameters					
Glucose (mmol/L)	4.82 ± 0.25 ^a^	5.54 ± 0.45 ^a^	6.82 ± 0.56 ^b^	7.21 ± 0.56 ^b^	0.018
Hepatic parameters					
TP (g/L)	5.01 ± 0.31	4.15 ± 0.24	4.81 ± 0.45	4.61 ± 0.49	0.449
Glucose (mmol/g prot)	3.92 ± 0.14 ^ab^	4.68 ± 0.35 ^b^	3.37 ± 0.65 ^ab^	3.25 ± 0.43 ^a^	0.101
GK (U/g prot)	0.90 ± 0.19	1.03 ± 0.15	1.35 ± 0.12	1.33 ± 0.19	0.197
PFKL (U/g prot)	5.54 ± 0.47 ^a^	6.68 ± 0.33 ^ab^	5.55 ± 0.57 ^a^	7.64 ± 0.96 ^b^	0.072
PK (U/g prot)	129.50 ± 15.22 ^a^	141.05 ± 5.05 ^a^	147.44 ± 21.32 ^a^	210.60 ± 26.49 ^b^	0.019
PC (U/g prot)	3.92 ± 0.24	4.43 ± 0.37	3.74 ± 0.35	4.93 ± 0.54	0.160
PEPCK (U/g prot)	4.83 ± 1.04	5.14 ± 0.35	5.87 ± 0.41	5.29 ± 0.44	0.666
G6P (U/mg prot)	159.45 ± 19.02	180.16 ± 19.21	199.36 ± 14.43	179.87 ± 21.13	0.543

In the same column, values with different small letter superscripts mean significant difference (*p* < 0.05).

## Data Availability

The data that support the findings of this study are available from the corresponding author upon reasonable request.
